# Identifying physiological determinants of 800 m running performance using post-exercise blood lactate kinetics

**DOI:** 10.1007/s00421-024-05504-4

**Published:** 2024-05-18

**Authors:** Takuya Watanabe, Takeru Inaba, Cody R. van Rassel, Martin J. MacInnis, Katsuyuki Kakinoki, Hideo Hatta

**Affiliations:** 1 Department of Sports Sciences, The University of Tokyo, 3-8-1 Komaba, Meguro-ku, Tokyo 153-8902 Japan; 2 Faculty of Kinesiology, University of Calgary, Calgary, AB T2N 1N4 Canada; 3 BlueWych LLC, Atsugi, Kanagawa 228-0015 Japan

**Keywords:** Lactate kinetics, Lactate transport, Middle-distance running, Bi-compartment model, Glycolysis, Lactate oxidation

## Abstract

**Purpose:**

The aims of the present study were to investigate blood lactate kinetics following high intensity exercise and identify the physiological determinants of 800 m running performance.

**Methods:**

Fourteen competitive 800 m runners performed two running tests. First, participants performed a multistage graded exercise test to determine physiological indicators related to endurance performance. Second, participants performed four to six 30-s high intensity running bouts to determine post-exercise blood lactate kinetics. Using a biexponential time function, lactate exchange ability (*γ*_1_), lactate removal ability (*γ*_2_), and the quantity of lactate accumulated (QLaA) were calculated from individual blood lactate recovery data.

**Results:**

800 m running performance was significantly correlated with peak oxygen consumption (r = −0.794), *γ*_1_ and *γ*_2_ at 800 m race pace (r = −0.604 and −0.845, respectively), and QLaA at maximal running speed (r = −0.657). ${\dot{\text{V}}}$O_2peak_ and *γ*_2_ at 800 m race pace explained 83% of the variance in 800 m running performance.

**Conclusion:**

Our results indicate that (1) a high capacity to exchange and remove lactate, (2) a high capacity for short-term lactate accumulation and, (3) peak oxygen consumption, are critical elements of 800 m running performance. Accordingly, while lactate has primarily been utilized as a performance indicator for long-distance running, post-exercise lactate kinetics may also prove valuable as a performance determinant in middle-distance running.

**Supplementary Information:**

The online version contains supplementary material available at 10.1007/s00421-024-05504-4.

## Introduction

The 800 m running event consists of supramaximal intensities of exercise (i.e., exceeding the minimal speed required to elicit 100% of maximal oxygen consumption (${\dot{\text{V}}}$O_2max_)) and requires rapid adenosine triphosphate (ATP) production from both glycolytic and oxidative processes in the skeletal muscle (Hill 1999; Spencer and Gastin 2001). Accordingly, a range of training strategies are necessary to enhance oxygen delivery and mitochondrial respiration, as well as physiological processes related to lactate production and oxidation (Brandon 1995). Whereas the maximal rate that oxygen can be consumed (i.e., ${\dot{\text{V}}}$O_2max_), the oxygen consumption (${\dot{\text{V}}}$O_2_) associated with the lactate threshold (LT), and the energy cost of running at a given speed are important determinants of long-distance running performance (Bassett and Howley 2000), their relevance to 800 m running performance is less obvious. Nevertheless, for 800 m runners, it is still important to quantify a runner’s physiological capacities to guide exercise training and develop effective racing strategies. In comparison to endurance running, quantifying the relative contribution of glycolysis to ATP production during high intensity exercise is more important (Maciejewski et al. 2013; del Arco et al. 2022), and the underlying determinants of supramaximal running performance, like the 800 m running event, are less established. Previously, the accumulated oxygen deficit (AOD) (Medbø et al. 1988), has been a widely accepted method to evaluate anaerobic ATP production during high intensity exercise (Gastin 2001); however, limitations of this method have been identified (Bangsbo 1996, 1998), and the validity of this estimation remains in question. The magnitude of AOD, for example, was reported to be affected by factors other than anaerobic energy production (Muniz-Pumares et al. 2017).

In addition to the AOD method, ATP production by the glycolytic system can be estimated by measuring lactate accumulation in muscle and blood. Peak blood lactate concentration ([BLa]) following exercise, for example, has been proposed as a metric related to anaerobic energy contribution (Bishop et al. 2002; Mero 1988). Despite its relationship with anaerobic energy supply, blood lactate accumulation is influenced by several factors, including the rates of lactate production, exchange, and consumption, all of which must be considered to derive meaning from lactate measurements (Maciejewski et al. 2013; Watanabe et al. 2023). Considering these factors, Freund and Gendry (1978) delineated the distribution of lactate in the organism following short-term strenuous exercise with an open two-compartment model comprised of the previously working muscles space and the remaining lactate space. Using this model, individual [BLa] recovery curves were fitted with a biexponential time function to identify abilities of lactate exchange between the previously active muscles and the blood (*γ*_1_) and removal from the organism (*γ*_2_) (Freund et al. 1986) and the quantity of lactate accumulated (QLaA), which represented the mobilization of glycolysis during exercise (Maciejewski et al. 2013; Bret et al. 2013; Chatel et al. 2016; Shirai et al. 2018). These indices have been shown to be related to fitness (Messonnier et al. 2001, 2006; Thomas et al. 2012, 2005), endurance performance (Bret et al. 2003; Messonnier et al. 1997), and physiological factors that affect endurance performance, such as the monocarboxylate transporter (Maciejewski et al. 2016, 2020), citrate synthase (CS) activity (Maciejewski et al. 2020), mitochondrial respiration (Thomas et al. 2004), and muscle fiber composition (Messonnier et al. 2002). Despite these findings, the relationship between indices of blood lactate kinetics and 800 m performance need to be further explored. Previously, Bret et al. (2003) identified a significant relationship between lactate exchange ability and 800 m performance, but the participants also included athletes specializing in sprint distances and the 1500 m, and these athletes may differ from 800 m runners (Crowther et al. 2002). Also, they have not considered the relationship between QLaA and performance.

The aim of the present study was to investigate the relationship between physiological indicators of both high intensity and endurance exercise with 800 m running performance The physiological indicators investigated were *γ*_1_, *γ*_2_, and QLaA, following a short-duration high intensity exercise test, and the running speed at lactate threshold (vLT), running speed at onset of blood lactate accumulation (vOBLA), and peak oxygen consumption (${\dot{\text{V}}}$O_2peak_) derived from a graded exercise test. In this manuscript, we use the term of LT as the first lactate threshold, whereas OBLA indicates the second lactate threshold. Using these physiological indicators of high intensity and endurance fitness, we then determined the best model to explain 800 m running performance. This modeling may provide a new evaluation method of the glycolytic system during 800 m running to help build effective physiologically driven training programs by identifying the factors contributing to 800 m running performance. We hypothesized that high lactate exchange and removal abilities, high short-term lactate accumulation, and high running speeds at the LT would be associated with the fastest 800 m running performances.

## Materials and methods

### Participants

Fourteen male runners (mean ± SD; age 25 ± 5 years; body mass 61.9 ± 4.1 kg; height 175.0 ± 4.6 cm) were recruited for this study based on a priori sample size determination. To calculate sample size, we used previously published results (Messonnier et al. 1997; Freund et al. 1986) that determined a significant relationship between post-exercise blood lactate kinetics with exercise performance and intensity, where correlation coefficients distributed approximately between 0.7 and 0.9. Assuming that these parameters are also highly correlated with 800 m running performance in the present study, a minimum sample size of 13 participants was computed based on a significance level of 0.05, a statistical power of 0.8, and a correlation coefficient of 0.7. All participants were previously competing in the 800 m running event for at least five years and training at least four times a week for the past two years. All participants had experience with treadmill running. The nature and risk of the experimental procedures were fully explained to all participants before providing written informed consent. This study was approved by the Research Ethics Committee of the University of Tokyo (No.689–3) and performed in accordance with the Declaration of Helsinki.

### Experimental design

Participants performed two exercise sessions in sequence consisting of a multistage graded exercise test (GXT) and a short-duration high intensity running test. The sessions were separated by at least 48 h. Prior to the testing sessions, participants were asked to refrain from intense exercise for at least 48 h, eating or drinking anything other than water for at least 3 h, and consuming caffeine on the same day as the test.

All testing procedures were performed on a motorized treadmill (T.K.K. 1255, Takei Scientific Instruments Co., Ltd., Japan) with an incline set to 0% gradient. Participants wore the same flat (i.e., non-spiked) running shoes normally worn during their high intensity training. For all tests, [BLa] was measured using the Lactate Pro 2 (LT-1730, ARKRAY Inc., Japan).

#### Session 1: Multistage graded exercise test

Prior to beginning the GXT, participants measured their resting [BLa] and performed self-directed warm-up exercises. The test began at a running speed of either 10, 11, or 12 km/h, selected based on each participant’s estimated fitness so that approximately nine stages were performed before test termination (Jamnick et al. 2018; Alves et al. 2017). Each running stage was 180 s followed by 60 s of standing rest, with running speed increased by 1 km/h for each subsequent stage. During each rest period, [BLa] and rating of perceived exertion (RPE), via the Borg (6–20) RPE scale (Borg 1982), were measured. The test was terminated when participants self-determined that they could not complete a subsequent stage. No participants stopped running in the middle of the 3-min stages.

Ventilatory and gas exchange variables were measured during the GXT using a metabolic cart (Aeromonitor AE-310S, Minato Medical Science Co., Ltd., Japan) connected to the expiration valve of a facemask (7450 Series V2, Hans-Rudolph, USA). Ventilatory and gas exchange data were calculated as 5-s averages. The metabolic cart system was calibrated using a 2 L syringe and gas mixture of known composition (4.976% CO_2_, 15.010% O_2_, and N_2_ for the balance).

#### Session 2: Short-term high intensity running test

After resting [BLa] measurements, participants performed self-directed warm-up exercises prior to the short-term high intensity running test. This test consisted of four to six 30-s running bouts at the running speed associated with peak oxygen consumption (v${\dot{\text{V}}}$O_2peak_) and at running speeds 2, 4, 6, 8, and 10 km/h faster than v${\dot{\text{V}}}$O_2peak_, performed in a random order. The 30-s duration for each bout of the high intensity running test was selected based on previous recommendations (Heck et al. 2003) to elicit an exercise duration in which maximal rates of lactate formation could be evaluated while avoiding information loss about lactate exchange due to saturation of [BLa] when the exercise duration is too long. If participants did not complete 30-s of running at any particular running speed, the run was omitted. [BLa] measurements were taken at 0.25, 0.5, 1, 1.5, 2, 2.5, 3, 3.5, 4, 4.5, 5, 6, 8, 10, 12, 15, 20, 25, 30, 40, 50, and 60 min after completing each running bout or until (1) post-exercise [BLa] dropped below 2.0 mmol/L, or (2) [BLa] measurements approached resting values but did not decrease by more than 10% for 20 min or longer. In 11 out of 66 number of cases, participants’ [BLa] did not drop below 2.0 mmol/L by 60 min following 30-s running bouts; however, the duration of time between subsequent running bouts was extended until [BLa] were confirmed to be less than 2.0 mmol/L. If desired, participants were allowed to perform additional self-directed warm-up exercises prior to conducting subsequent 30-s bouts. Due to time constraints, one participant underwent testing on two separate days.

### Data analysis

#### 800 m performance

Participant 800 m performance times were identified using their fastest 800 m running performance during an official competition within four months prior to or following the date in which the short-term high intensity running test was performed. For a participant who did not compete in an official competition during the study period (n = 1), the 800 m running performance from a competition closest to the second session of the test was used to indicate their 800 m performance time.

#### Graded exercise test analysis

The ${\dot{\text{V}}}$O_2_ (mL/kg/min) associated with each stage of the GXT was identified as the highest 30-s average ${\dot{\text{V}}}$O_2_ at each respective stage. Peak oxygen uptake (${\dot{\text{V}}}$O_2peak_, mL/kg/min) was determined as the highest 30-s average ${\dot{\text{V}}}$O_2_ during the GXT. The running speed at which ${\dot{\text{V}}}$O_2peak_ was recorded was defined as v${\dot{\text{V}}}$O_2peak_ (km/h). The running speed at LT (vLT, km/h) was defined as the speed at which [BLa] increased by 1 mmol/L above baseline (Coyle et al. 1983). The running speed at onset of blood lactate accumulation (vOBLA, km/h) was defined as the speed at which [BLa] was 4.0 mmol/L calculated by linear interpolation.

#### Short-term high intensity test analysis

**Fig. 1:**
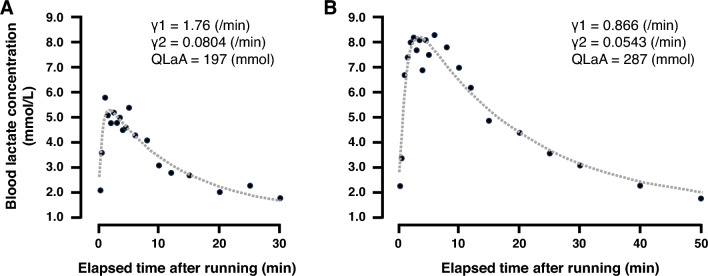
Representative blood lactate recovery curves following the 30-s high intensity running bout performed at 24 km/h from the participant with the fastest 800 m performance (1 min 49 s; **A**) and slowest 800 m performance (2 min 07 s; **B**), respectively. The dotted lines represent the biexponential time function fit to each participant’s post-exercise blood lactate measurements (black circles) to calculate lactate exchange ability (*γ*_1_), lactate removal ability (*γ*_2_), and quantity of lactate accumulated (QLaA)

Individual post short-term high intensity exercise [BLa] data were fit using the following biexponential time function proposed by Freund and Gendry (1978):

1 $$\begin{array}{c}La\left(t\right)=La\left(0\right)+{A}_{1}\left(1-{\text{exp}}\left(-{\gamma }_{1}t\right)\right)+{A}_{2}\left(1-{\text{exp}}\left(-{\gamma }_{2}t\right)\right)\end{array}$$

where La(t) and La(0) (mmol/L) are the [BLa] at time t and at the onset of recovery, respectively; *γ*_1_ and *γ*_2_ (/min) are the velocity constants representing lactate exchange and removal, respectively; and *A*_1_ and *A*_2_ (mmol/L) representing their respective amplitudes. To accurately calculate the parameters of this model, post-exercise [BLa] measures must reach 2.0 mmol/L so that the function does not converge at [BLa] values higher than resting values. Therefore, in 11 cases when [BLa] did not drop below 2.0 mmol/L by 60 min following a 30-s high-intensity exercise bout, [BLa] recovery curves were calculated based on the assumption that [BLa] would return to resting values 140 min after each bout to achieve a more accurate recovery curves (Maciejewski et al. 2013; Durand et al. 2021). Parameters were calculated using a least-squares regression model implemented by the optimize.leastsq function from the SciPy 1.10.1 package and Python (ver. 3.11.3, Python Software Foundation, USA) coding language. Figure 1 shows examples of fitting [BLa] curve for individual [BLa] change.

The quantity of lactate accumulated (QLaA, mmol) was calculated as the summation between peak lactate accumulation (i.e., QLaA_peak_, mmol) and the quantity of lactate removed between the onset of recovery until peak lactate accumulation (i.e., QLaR, mmol) as follows:

2 $$\begin{array}{c}QLaA={QLaA}_{peak}+QLaR\end{array}$$

3 $$\begin{array}{c}{QLaA}_{peak}={La}_{peak}\cdot {V}_{TLS}\end{array}$$

4 $$\begin{array}{c}QLaR=\frac{{La}_{peak}+La\left(0\right)}{2}\cdot {\gamma }_{2}\cdot t{La}_{peak}\cdot {V}_{TLS}\end{array}$$

where La_peak_ (mmol/L) is peak blood lactate concentration; V_TLS_(L) is the volume of the total lactate distribution space, calculated as 500 mL/kg body mass (Bret et al. 2013; Chatel et al. 2016); and tLa_peak_ (min) is the time to reach La_peak_. Since the time derivative of [BLa] at peak concentration was equal to zero, La_peak_ and tLa_peak_ can be calculated as follows:

5 $$\begin{array}{c}{tLa}_{peak}=\frac{1}{{\gamma }_{1}-{\gamma }_{2}}{\text{ln}}\left(-\frac{{A}_{1}{\gamma }_{1}}{{A}_{2}{\gamma }_{2}}\right)\end{array}$$

6 $$\begin{array}{c}{La}_{peak}=La\left(t{La}_{peak}\right).\end{array}$$

**Fig. 2:**
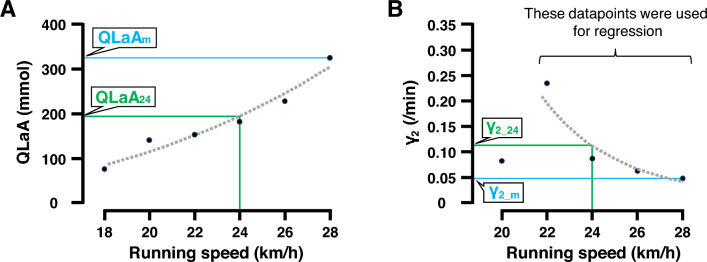
Representative curves for analysis of lactate kinetics data. Panel A shows an example lactate accumulation (QLaA) response following the high intensity running test, from one participant. QLaA_m_, indicated by the blue line, was identified as the QLaA associated with the participant’s fastest achieved running speed (i.e., 28 km/h). QLaA_24_, indicated by the green line, was calculated by extrapolation from a log-linear regression curve (dotted line) using all QLaA responses from the running test to indicate the QLaA at 24 km/h. Panel B shows an example lactate removal ability (*γ*_2_) responses for a different participant following the high intensity running test. The *γ*_2_m_, indicated by the blue line, was identified as the *γ*_2_ associated with the participant’s fastest achieved running speed. The *γ*_2_24_, indicated by the green line, was calculated by extrapolation using γ_2_ data points between the maximum value (i.e., at 22 km/h) and value at the fastest running speed (i.e., 28 km/h). In this case, the γ_2_ value at 20 km/h was excluded from the regression calculation, as this intensity was low to evaluate the relationship between lactate removal ability and running intensity. Lactate exchange ability at 24 km/h (*γ*_1_24_) and lactate exchange ability at the maximal running speed (*γ*_1_m_) were calculated using the same procedures

Maximal QLaA_m_ (mmol), γ_1_m_ (/min), and γ_2_m_ (/min) parameters were calculated using the blood lactate response following the fastest running speed completed by each participant during the short-term high intensity running test. Alternatively, the fixed 24 km/h QLaA_24_ (mmol), γ_1_24_ (/min), and γ_2_24_ (/min) were calculated by extrapolation using a log-linear regression of the QLaA, γ_1_, and γ_2_ responses from all the running test speeds performed by each participant. For QLaA_24_ calculation, all QLaA responses were included in the regression (Fig. 2A). For γ_1_24_, and γ_2_24_ determination, only the γ_1_ and γ_2_ values between the maximum value and value at the fastest running speed were used for regression, as the γ_1_ and γ_2_ responses may increase prior to decreasing with an increase in exercise intensity displaying an upward convex trend (Fig. 2B) (Freund et al. 1989). The 24 km/h running speed was selected for comparison because this speed was determined to most closely match the 800 m pace associated with the average participant 800 m running performance (i.e., 118.23 ± 6.08 s). However, not all runners performed the 24 km/h bout (n = 6) so an estimation of QLaA_24_, γ_1_24_, and γ_2_24_ using the existing parameters was necessary.

### Statistical analysis

Sample size calculations were performed using G*power software (version 3.1.9.6, Franz Faul, Universität Kiel, Germany). All subsequent statistical analyses were performed using R software (version 4.3.0, The R Foundation for Statistical Computing, Austria). Pearson correlation coefficients were used to determine the correlation between physiological parameters and 800 m performance. To determine which physiological variables were most predictive of 800 m running performance, simple and multiple linear regression analyses were conducted using 800 m running performance times as dependent variables and ${\dot{\text{V}}}$O_2peak_, vLT, vOBLA and lactate kinetics parameters (i.e., QLaA_m_, QLaA_24_, *γ*_1_m_, *γ*_1_24_,* γ*_2_m_, and *γ*_2_24_) as independent variables. When the sample size used to construct a mathematical model is relatively small (i.e., n = 14) compared to the number of independent variables (i.e., 9), the model might experience overfitting. Based on previous recommendations, the number of independent variables should be less than one-tenth of the sample size (Harrell 2015). Therefore, multiple linear regression models were limited to using one and two independent variables. The best 800 m running performance prediction model was determined as the regression model with the smallest Akaike’s information criterion (Akaike 1974). To assess multicollinearity among variables, the variance inflation factor (VIF) was calculated using the R package MASS (version 7.3.60). Data visualization was performed using Microsoft PowerPoint (version 2401, Microsoft Corporation, USA) for Figs. 1 and 2, and Prism (version 10.1.0, GraphPad Software, USA) forthe other figures. The data are reported as mean ± standard deviation (SD). The level of statistical significance was set at α < 0.05.

## Results

Participants recent 800 m running performances, GXT results, and blood lactate kinetic parameters are noted in Table 1. During the GXT, participants completed 9 ± 1 stages and the average running speed during the final stage was 19 ± 1 km/h. During the short-term high intensity running test, participants performed 5 ± 1, 30-s running bouts on average and achieved an average maximal running speed of 26 ± 2 km/h. Post-exercise peak [BLa] at 24 km/h and at maximal running speed were 6.24 ± 1.54 (n = 8) and 9.38 ± 3.23 mmol/L (n = 14), respectively.

**Table 1:** Participant 800 m running performance, graded exercise test, and short-term high intensity running test results

Exercise Test	Variables	Mean ± SD
Official competition	800 m performance (sec)	118.23 ± 6.08
Session 1: Multistage graded exercise test	vLT (km/h)	15.6 ± 0.9
	vOBLA (km/h)	16.2 ± 1.1
	${\dot{\text{V}}}$O_2peak_ (ml/min/kg)	63.4 ± 6.0
Session 2: Short-term high intensity running test	QLaA_m_ (mmol)	324.4 ± 109.5
	QLaA_24_ (mmol)	221.7 ± 31.3
	γ_1_m_ (/min)	1.042 ± 0.342
	γ_1_24_ (/min)	1.402 ± 0.618
	γ_2_m_ (/min)	0.0622 ± 0.0207
	γ_2_24_ (/min)	0.0897 ± 0.0358

vLT running speed at lactate threshold; vOBLA running speed at onset of blood lactate concentration; ${\dot{\text{V}}}$O_2peak_ peak oxygen consumption; QLaA_m_ quantity of lactate accumulated at maximal running speed; QLaA_24_ quantity of lactate accumulated at 24 km/h; γ_1_m_ velocity constant representing lactate exchange ability at maximal running speed; γ_1_24_ velocity constant representing lactate exchange ability at 24 km/h; γ_2_m_ velocity constant representing lactate removal ability at maximal running speed; γ_2_24_ velocity constant representing lactate removal ability at 24 km/h

### Lactate kinetics parameters and running speed

**Fig. 3:**
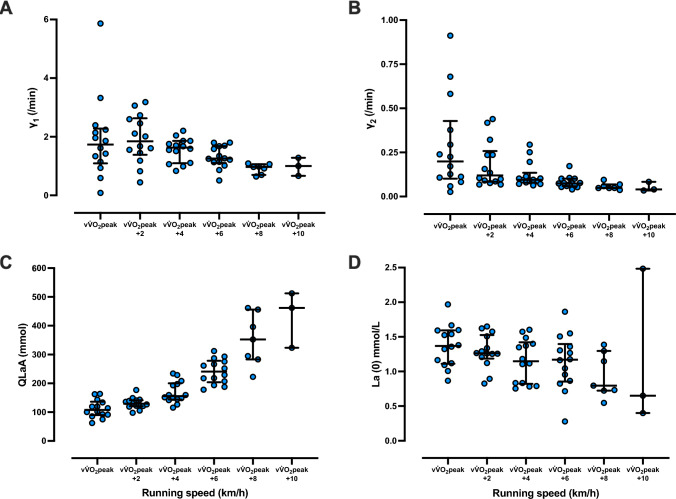
Lactate kinetics parameters for running speeds relative to the speed at peak oxygen consumption (v${\dot{\text{V}}}$O_2peak_). **A**–**D** represent lactate exchange ability (γ_1_), lactate removal ability (γ_2_), quantity of lactate accumulated (QLaA) and blood lactate concentration at the onset of recovery (La(0)) derived from the short-term high intensity running tests performed at the running speed associated with v${\dot{\text{V}}}$O_2peak_ and at running speeds 2, 4, 6, 8 and 10 km/h faster than v${\dot{\text{V}}}$O_2peak._ Individual data points are presented with central bars representing the median and error bars representing the interquartile range. All participants completed running trials at v${\dot{\text{V}}}$O_2peak_ and v${\dot{\text{V}}}$O_2peak_ + 2, 4, and 6 km/h (i.e., n = 14). However, seven participants were not able to complete running trials at v${\dot{\text{V}}}$O_2peak_ + 8 km/h (n = 7), and 11 participants were not able to complete running trials at v${\dot{\text{V}}}$O_2peak_ + 10 km/h (n = 3)

The relationships between running speed (relative to v${\dot{\text{V}}}$O_2peak_) and lactate kinetics parameters, γ_1_, γ_2_, QLaA, and La(0) are presented in Fig. 3. The individual changes in each parameter are provided in Supplemental Tables 1–4.

### The correlation coefficient with 800 m performance and other variables

**Fig. 4:**
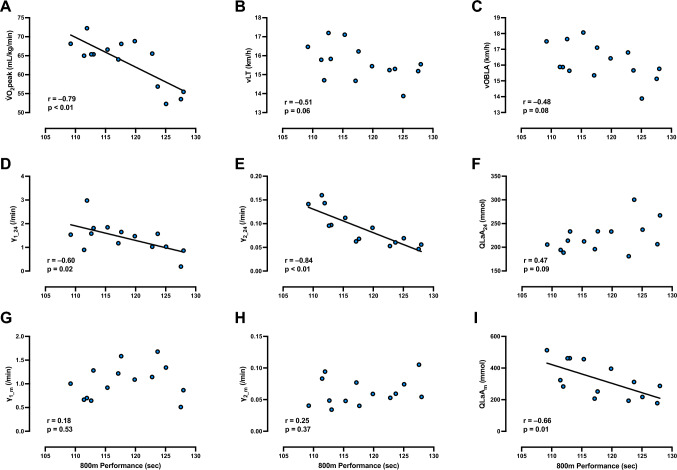
Relationships between 800 m running performance and variables obtained from a graded exercise test and multiple short-duration, high intensity runs. **A–I** depict the relationship between 800 m running performance with **A** peak oxygen consumption (${\dot{\text{V}}}$O_2peak_), **B** running speed at lactate threshod (vLT), **C** running speed at onset of blood lactate concentration (vOBLA), **D** lactate exchange ability at 24 km/h (γ_1_24_), **E** lactate removal ability at 24 km/h (γ_2_24_), **F** quantity of lactate accumulated at 24 km/h (QLaA_24_), **G** lactate exchange ability at maximal running speed (γ_1_m_), **H** lactate removal ability at maximal running speed (γ_2_m_), and **I** quantity of lactate accumulated at maximal running speed (QLaA_m_), respectively. n = 14 for all panels

**Fig. 5:**
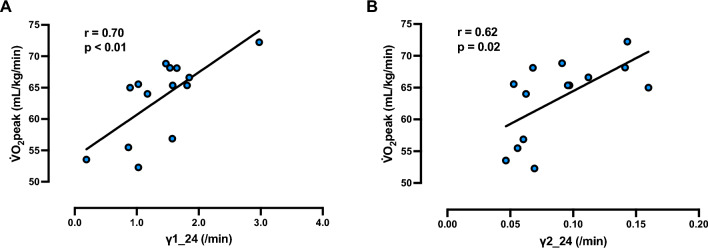
Relationships between peak oxygen consumption (${\dot{\text{V}}}$O_2peak_) and lactate kinetics parameters. **A**, **B** represent scatter plots between ${\dot{\text{V}}}$O_2peak_ with lactate exchange ability at 24 km/h (γ_1_24_) and lactate removal ability at 24 km/h (γ_2_24_), respectively. n = 14 for all panels

**Fig. 6:**
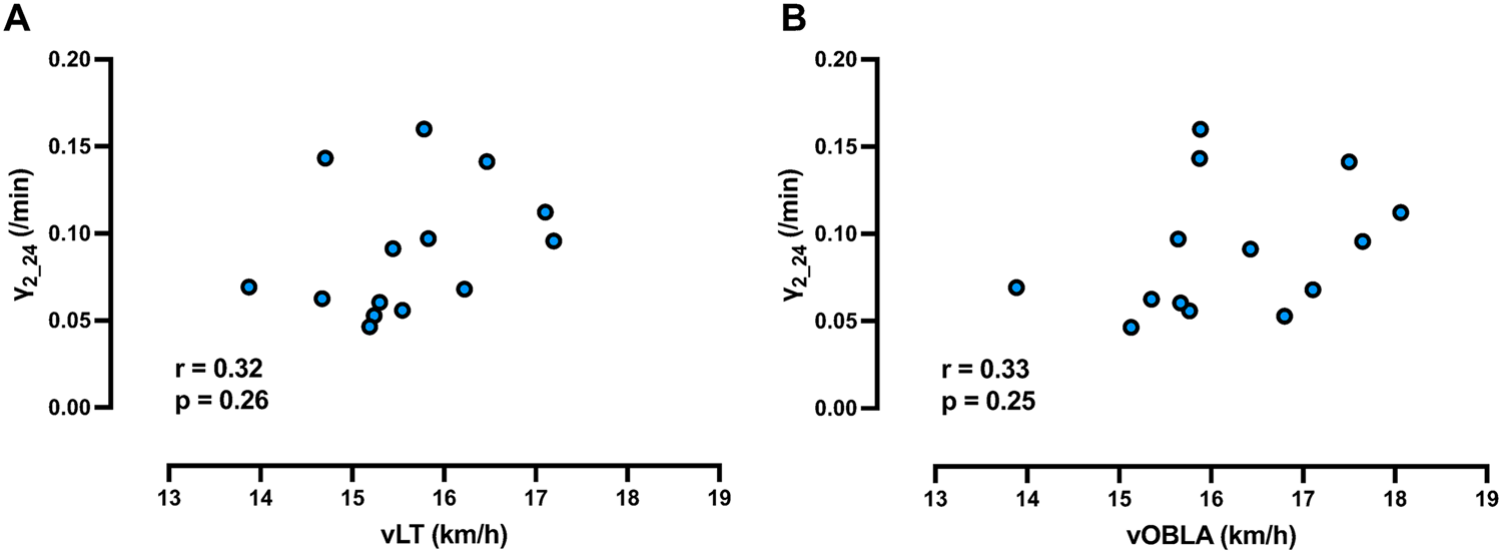
Relationships between lactate removal ability at 24 km/h (γ_2_24_) with running speed at lactate threshod (vLT) and at onset of blood lactate concentration (vOBLA). **A**, **B** represent scatter plots between γ_2_24_ with vLT and vOBLA, respectively. n = 14 for all panels

The relationship between 800 m performance and ${\dot{\text{V}}}$O_2peak_, vLT, vOBLA, γ_1_24_, γ_2_24_, QLaA_24_, γ_1_m_, γ_2_m_ and QLaA_m_ are presented in Fig. 4. ${\dot{\text{V}}}$O_2peak_ and γ_2_24_ were strongly correlated with 800 m performance, while QLaA_m_ and γ_1_24_ were moderately correlated with 800 m running performance. No significant correlations were detected between the other variable pairings and 800 m running performance. The relationship between ${\dot{\text{V}}}$O_2peak_ with γ_1_24_ and γ_2_24_ are presented in Fig. 5. γ_1_24_ and γ_2_24_ were correlated not only with 800 m performance but also with ${\dot{\text{V}}}$O_2peak_. No significant correlations were detected between γ_2_24_ with either vLT or vOBLA (Fig. 6).

### 800 m running performance prediction model

**Fig. 7:**
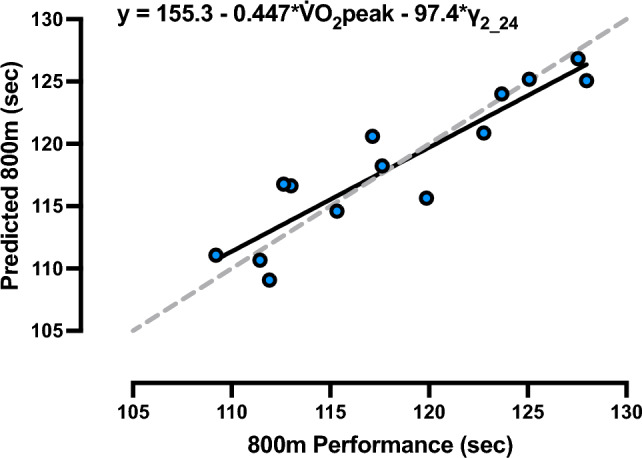
Regression between the predicted and measured 800 m running performance in seconds. Units of peak oxygen consumption (${\dot{\text{V}}}$O_2peak_) and lactate removal ability at 24 km/h (γ_2_24_) are in mL/kg/min and /min, respectively. The dotted line represents line of identity. n = 14

The results of multiple linear regression analysis for predicting 800 m performance using physiological variables are presented in Table 2. The independent variables selected for the model were ${\dot{\text{V}}}$O_2peak_ and γ_2_24_. The standardized regression coefficient of γ_2_24_ (0.57) was greater than that of ${\dot{\text{V}}}$O_2peak_ (0.44) in absolute value. The p-value of the overall model was significant, with a high degree-of-freedom adjusted coefficient of determination at 0.80. These two variables could explain 83% of the variance in 800 m performance (Fig. 7). The *p*-values of each variable and the intercept were also significant, and the VIF was less than 10, confirming the absence of multicollinearity.

**Table 2:** The final determined linear regression model used to predict 800 m running performance. For 800 m running performance (sec) =  − 0.4465 × ${\dot{\text{V}}}$O_2peak_ (mL/kg/min) − 97.42 × γ_2_24_ (/min) + 155.3

Variables	Regression coefficient	Standardized regression coefficient	t-value	*p*-value	VIF
(intercept)	155.3	–	17.69	< 0.001	–
${\dot{\text{V}}}$O_2peak_ (mL/kg/min)	− 0.4465	− 0.4408	− 2.828	0.016	1.612
γ_2_24_ (/min)	− 97.42	− 0.5732	− 3.678	0.004	1.612

Model statistics F-value: 27.68; *p*-value: < 0.001; R^2^: 0.8342; Adjusted R^2^: 0.8041

${\dot{\text{V}}}$O_2peak_ peak oxygen consumption; γ_2_24_ lactate removal ability at 24 km/h; VIF variance inflation factor

## Discussion

The results from our investigation provide evidence that 800 m running performance is determined by both oxidative and non-oxidative glycolytic processes. Specifically, a high capacity to exchange and remove lactate at the running speed associated with the 800 m running event (i.e., 24 km/h), a high capacity for short-term lactate production, and high maximal rates of oxygen consumption (i.e., ${\dot{\text{V}}}$O_2peak_) were all associated with enhanced 800 m running performances. Among them, lactate removal abilities at the running speed associated with the 800 m running event and ${\dot{\text{V}}}$O_2peak_ had the strongest associations with middle-distance running performance times. These findings imply that concurrent enhancement of central cardiopulmonary function (i.e., oxygen delivery) and the metabolic capacity of peripheral muscles and organs (e.g., the efficiency of lactate oxidation) are essential for maximizing 800 m running performance.

### Post-exercise blood lactate exchange (γ_1_) and removal (γ_2_) abilities

There appears to be an effect of running speed on lactate exchange and removal abilities at an individual level, as the range in γ_1_ and γ_2_ responses narrowed and the values seemed to display slight reductions with increases in running speed (Fig. 3A, B). This reduction in lactate exchange and removal abilities with exercise intensity is similar to previous studies (Freund et al. 1986, 1989). Our data suggests that low lactate exchange and removal abilities at lower intensities close to v${\dot{\text{V}}}$O_2peak_ may be related to poor fitting curves by low lactate production and accumulation. This is why γ_1_24_ and γ_2_24_ were not calculated by using all data points (Fig. 2B). These findings suggest that lactate management responses during supramaximal exercise are dependent on exercise intensity and are subject specific. Interindividual differences in lactate exchange and removal abilities were also detected at the running speed associated with the 800 m running event (i.e., 24 km/h). At this intensity, runners who maintained relatively high lactate exchange and removal abilities (i.e., γ_1_24_ and γ_2_24_) were also faster 800 m runners (Fig. 4D, E) supporting our hypothesis that lactate exchange and removal abilities influence supramaximal exercise performance. Despite these findings, lactate exchange and removal abilities seemed to reach a nadir in all runners as running speed approached the maximal speed that could be maintained for 30 s (Fig. 3A, B). Together, our findings indicate that lactate exchange and removal handling likely vary between individuals, that these processes are dependent upon running intensity, and that maintaining high lactate exchange and removal abilities at supramaximal intensities can lead to improved middle-distance running performance.

The role that lactate exchange (shuttling and delivery of lactate from the previously working musclesinto the blood), and lactate removal (primarily via oxidation in the muscle and cardiac tissue for ATP provision), have on exercise performance has been widely investigated. For example, lactate exchange and removal abilities explained the majority of variance in rowing time trial performance (Messonnier et al. 1997) and were associated with improvements in fitness following 4 weeks of training (Messonnier et al. 2001). Similar to the present investigation, maximal lactate accumulation rate—a proxy measure of lactate exchange ability—was found to be positively correlated with 15-s all-out exercise (Quittmann et al. 2020) and 400 m time trial performance (Takei et al. 2018). Further, Bret et al. (2003) reported a significant correlation between 800 m running performance and lactate exchange ability, but in contrast to our investigation, no correlation was detected between 800 m performance and lactate removal ability following 1-min of constant speed running (i.e., 25.2 km/h). The reason for this discrepancy might be due to the heterogeneity in their study population, which consisted of 1500 m runners, 800 m runners, and sprinters (Bret et al. 2003). Due to the considerable differences in muscle fiber type between sprinters and middle-distance runners (Costill et al. 1976), along with the faster glycogen resynthesis rates in fast-twitch muscle fibers compared to slow-twitch muscle fibers (Vøllestad et al. 1989), differences in lactate kinetics between sprinters and middle-distance runners may have led to the non-significant lactate exchange findings (Taoutaou et al. 1996; Takei et al. 2018). In contrast, the present study exclusively recruited trained 800 m runners, which may be why a significant relationship between lactate exchange ability and 800 m performance was detected.

Lactate exchange and removal rates at maximal running speeds were not related to 800 m performance, which may be expected, as these relationships were assessed at running speeds that were neither the same absolute nor relative intensity (Fig. 4G, H).

### Post-exercise lactate accumulation (QLaA)

Our results highlight the strong relationship between lactate accumulation and 800 m running performance. As expected, post-exercise lactate accumulation (i.e., QLaA) increased as relative supramaximal intensity increased (Fig. 3C), and runners who were able to produce the largest quantities of lactate following maximal sprint bouts (i.e., QLaA_m_) were also found to have the fastest 800 m running performances (Fig. 4I). Post-exercise lactate accumulation can be regarded as an indicator of how much energy is produced during 30-s of all-out exercise by the glycolytic system. Accordingly, these results indicate that greater maximal lactate accumulation, and thus, greater maximal capacities of glycolytic energy production are associated with middle-distance running performance.

The strong relationship found between QLaA_m_ and 800 m performance is similar to previous investigations that detected relationships between short-distance sprint performance and 800 m performance (Bachero-Mena et al. 2017; Deason et al. 1991; Støren et al. 2021). During these investigations, [BLa] was not evaluated; however, the significant relationship between [BLa] after the 200 m race and race speed has been reported (Hautier et al. 1994). Sprint performance is generally related to the proportion of available fast-twitch muscle fibers, the capacity to activate high threshold fast-twitch motor units, rate of force development, and maximal forces applied to the ground (Colliander et al. 1988; Bachero-Mena et al. 2017; Weyand et al. 2000). Accordingly, the relationship between QLaA_m_ and 800 m running performance in the present study may indicate that faster middle-distance runners also had greater proportions of fast-twitch fibers, and/or had a greater capacity to mobilize high threshold motor units.

In contrast to QLaA_m_, lactate accumulation at 24 km/h (QLaA_24_) was not significantly correlated with 800 m performance (Fig. 4F). Lactate accumulation at a certain speed can be considered as an indicator of how much the glycolytic system is activated at any given exercise intensity. The results of the present study may indicate that the effects of glycolysis and the greater accumulation of lactate during short-term at a certain high intensity do not have a negative effect on performance. The assertion by previous studies that lactate is not a direct cause of fatigue and multiple deleterious effects (Karlsson et al. 1975) is consistent with the results of the present study.

### ${\dot{\text{V}}}$O_2peak_, vLT, and vOBLA

The results from the GXT suggest that ${\dot{\text{V}}}$O_2peak_ is more reflective of middle-distance running performance than vLT and vOBLA, with a strong negative correlation found between ${\dot{\text{V}}}$O_2peak_ and 800 m running performance time (i.e., runners with higher ${\dot{\text{V}}}$O_2peak_ had faster performance times; Fig. 4A). Previous investigations have also suggested that ${\dot{\text{V}}}$O_2max_ plays a substantial role in the success of the 800 m running performance (Brandon 1995; Ingham et al. 2008; Nevill et al. 2008), with high ${\dot{\text{V}}}$O_2max_ found to be related to lactate transport capacity (Pilegaard et al. 1994). The results of the present study also showed a significant correlation between ${\dot{\text{V}}}$O_2peak_ and γ_1_24_ (Fig. 5A) and γ_2_24_ (Fig. 5B), which does not contradict the results and interpretations of previous studies (Pilegaard et al. 1994; Messonnier et al. 1997).

Contrary to our hypothesis, no significant correlations were detected between 800 m performance and vLT (Fig. 4B) or vOBLA (Fig. 4C). Although significant correlation between vLT and 800 m performance have previously been reported, the correlation was only moderate (i.e., r = 0.49) (Ingham et al. 2008). Typically, the vLT and vOBLA are measurements obtained from an incremental exercise used to estimate the upper limits of sustainable exercise performance during long-distance events (Hill 1999). In contrast to ${\dot{\text{V}}}$O_2max_, exercise performance at the vLT and vOBLA depend more upon oxygen extraction, mitochondria content, and slow-twitch muscle fiber content. Accordingly, these results imply that 800 m running performance is dictated by improvement in maximal rates of oxygen delivery from cardiovascular adaptations. Together, these results support potential reasons why 800 m runners tend to have higher percentages of fast-twitch fibers compared to long-distance runners (Costill et al. 1976). Notably, glycolysis is more likely to be activated in fast-twitch fibers. Furthermore, some previous studies have reported significant correlations between lactate removal ability and the power corresponding to LT or OBLA (Oyono-Enguéllé et al. 1990; Messonnier et al. 1997), but no significant correlations between γ_2_24_ and vLT or vOBLA were detected in the present study (Fig. 6). Taken together, these facts suggest that vLT and vOBLA may not have adequately reflected the 800 m performance, unlike in longer distance events. Additionally, vOBLA was not significantly correlated with the 800 m performance may relate to the lack of a gold standard for assessing lactate curves (Jamnick et al. 2018) or the effects of diet and recent exercise on variability in [BLa] responses (Quinn et al. 2024).

### Prediction of 800 m running performance

The ${\dot{\text{V}}}$O_2peak_ measured by the GXT and lactate removal ability at 24 km/h obtained by the short-term high intensity running test were employed as the independent variables to explain the 800 m running performance. This result indicates that lactate removal ability had a larger contribution to 800 m performance than lactate exchange ability and is consistent with the results of a previous study (Messonnier et al. 1997). In addition, a previous investigation found that lactate production in the second half of the 2-min supramaximal exercise was significantly lower than in the first half (Kitaoka et al. 2014), suggesting that the contribution of energy production from non-oxidative glycolysis is smaller in the second half. Therefore, even in the 800 m run, which is an approximately 2-min exercise bout, mitochondrial ATP synthesis, including lactate oxidation, is more important than ATP synthesis from non-oxidative glycolysis in the second half of the race. In the 800 m run, how a runner deals with the slowdown in the last 200 m is a major performance determinant (Amo et al. 2021); therefore, it is reasonable to assume that the ability to remove lactate influences 800 m performance more than exchange. ${\dot{\text{V}}}$O_2peak_ is related to cardiovascular capacity, and lactate removal ability can be interpreted as encompassing factors that contribute to 800 m performance that cannot be explained by ${\dot{\text{V}}}$O_2peak_. In other words, the efficiency of lactate removal (e.g., lactate oxidation by peripheral muscles, gluconeogenesis, glycogen repletion, and other factors) may contribute to 800 m performance due to factors other than improved oxidative metabolic capacity. While interpreting the results, caution is warranted due to the significant correlation (r = 0.62, *p* = 0.019) between ${\dot{\text{V}}}$O_2peak_ and γ_2_24_. Nonetheless, it is believed that the concurrent enhancement of central cardiopulmonary function and the metabolic capacity of peripheral muscles can be effective in improving 800 m performance. These findings suggest that lactate kinetics parameters may be practical metrics for assessing 800 m performance, just as ${\dot{\text{V}}}$O_2_ and [BLa] during incremental exercise have been used to monitor longitudinal performance for long-distance athletes (Jones 2006).

### Limitations

The post-exercise blood lactate kinetics might be affected by the time delay between lactate production at the muscle and measurement in the blood. As lactate exchange processes begin in alignment with the onset of exercise, in particular, lactate exchange abilities are influenced by exercise duration. In an attempt to estimate lactate exchange abilities most precisely, the present study employed 30 s as the exercise duration, which is shorter than any protocols of previous studies that investigated post-exercise lactate kinetics. Therefore, it should be noted that simple comparisons to previous studies cannot be made regarding the value of the exchange and removal abilities. The La(0) estimated in the present study were 1.20 ± 0.38 mmol/L on average, generally distributed between 0.5 and 1.5 (Fig. 3D), and similar to day-to-day resting [BLa] measured at rest in a trained sprinter (i.e., 1.17 ± 0.34 mmol/L) (Kano and Sato 2021). The γ_1_ values obtained in the present study were greater than in the previous studies (Bret et al. 2013; Chatel et al. 2016) because of the short duration of exercise (Freund et al. 1989). As a result of focusing on measuring lactate exchange and removal abilities precisely, the protocol of the present study did not focus on lactate metabolism during an exercise lasting approximately 2 min, such as an 800 m run. To gain additional insights into lactate kinetics during an 800 m run, it is necessary to create a protocol that more closely simulates an 800 m race.

Finally, the range of coefficient of variation in lactate measurements from the lactate analyzer used in the present study is approximately 2–4%, with higher [BLa] producing larger SDs. Thus, measurement error from the lactate analyzer used in the present study may have significantly affected our results and more accurate [BLa] assessments and multiple measurements for each blood sampling assessment should be considered for future investigations.

## Conclusion

In summary, 800 m running performance was significantly correlated with the abilities to exchange and remove lactate at the running speed associated with 800 m racing, maximum lactate accumulation in a 30-s run, and ${\dot{\text{V}}}$O_2peak_. Lactate removal ability and ${\dot{\text{V}}}$O_2peak_ could explain 83% of the variance in 800 m performance. Therefore, from the perspective of lactate metabolism, (1) greater abilities to exchange and remove lactate, and (2) a high capacity for short-term lactate accumulation can contribute to improve 800 m performance. The multiple physiological and mechanical characteristics dictate 800 m performance (Bellinger et al. 2021), and the present study was the first to focus on post-exercise lactate kinetics to demonstrate this. Although lactate has primarily been utilized as a performance indicator for long-distance running, our results suggest that lactate kinetics can also be used as a performance indicator during short-duration high intensity exercises like middle-distance running. Moreover, measuring lactate kinetics parameters could be a practical method to inform training strategies and monitor 800 m potential.

## Supplementary Information

Below is the link to the electronic supplementary material.

Supplementary file1 (DOCX 59 KB)

## Data Availability

The data that support the findings of this study are available upon reasonable request from the corresponding author.

## Abbreviations

AOD: Accumulated oxygen deficit
ATP: Adenosine triphosphate
[BLa]: Blood lactate concentration
GXT: Multistage graded exercise test
La_peak_: Peak blood lactate concentration
La(0): Blood lactate concentration at the onset of recovery
LT: Lactate threshold
OBLA: Onset of blood lactate accumulation
QLaA: Quantity of lactate accumulated
QLaA_m_: QLaA at maximal running speed
QLaA_peak_: QLaA at peak blood lactate concentration
QLaA_24_: QLaA at 24 km/h
QLaR: Quantity of lactate removed from end of exercise to La_peak_
RPE: Rate of perceived exertion
SD: Standard deviation
tLa_peak_: Time to reach La_peak_
VIF: Variance inflation factor
vLT: Running speed at LT
vOBLA: Running speed at OBLA
$${\dot{\text{V}}}$$O_2_: Oxygen consumption
${\dot{\text{V}}}$O_2max_: Maximal oxygen consumption
${\dot{\text{V}}}$O_2peak_: Peak oxygen consumption
V_TLS_: Volume of the total lactate distribution space
v${\dot{\text{V}}}$O_2peak_: Running speed at peak oxygen consumption
γ_1_: Velocity constant representing lactate exchange ability
γ_1_m_: γ_1_ at maximal running speed
γ_1_24_: γ_1_ at 24 km/h
γ_2_: Velocity constant representing lactate removal ability
γ_2_m_: γ_2_ at maximal running speed
γ_2_24_: γ_2_ at 24 km/h
