# Supplementary material for: Identifying physiological determinants of 800 m running performance using post-exercise blood lactate kinetics
Source: Eur J Appl Physiol. 2024 May 18;124(10):2951–64. doi: 10.1007/s00421-024-05504-4 (PMC11467099; doi:10.1007/s00421-024-05504-4)
Supplement: Supplementary file 1 — Supplementary file1 (DOCX 59 KB) [file 421_2024_5504_MOESM1_ESM.docx]

**Supplemental Table 1** The individual changes in velocity constant representing lactate exchange ability (γ_1_, /min) for each running speed completed relative to peak oxygen consumption (vV̇O_2peak_)

| **Participant** | **vV̇O_2peak_** | **vV̇O_2peak_+2** | **vV̇O_2peak_+4** | **vV̇O_2peak_+6** | **vV̇O_2peak_+8** | **vV̇O_2peak_+10** |
| --- | --- | --- | --- | --- | --- | --- |
| 01 | 0.593 | 1.184 | 0.835 | 1.802 | 0.700 | - |
| 02 | 3.325 | 1.608 | 2.204 | 1.132 | 1.088 | - |
| 03 | 1.337 | 1.449 | 1.549 | 0.866 | - | - |
| 04 | 1.858 | 2.457 | 1.675 | 1.678 | - | - |
| 05 | 1.422 | 2.732 | 1.589 | 1.583 | - | - |
| 06 | 2.393 | 2.010 | 1.064 | 1.144 | - | - |
| 07 | 5.863 | 3.185 | 1.859 | 1.343 | - | - |
| 08 | 1.144 | 0.824 | 1.511 | 0.510 | - | - |
| 09 | 2.140 | 3.063 | 1.703 | 1.218 | - | - |
| 10 | 2.242 | 2.108 | 1.755 | 1.019 | 0.982 | 1.004 |
| 11 | 0.085 | 1.678 | 1.859 | 1.264 | 0.919 | - |
| 12 | 0.939 | 1.554 | 2.044 | 1.799 | 1.064 | 1.282 |
| 13 | 1.615 | 2.599 | 1.108 | 1.696 | 0.644 | - |
| 14 | 1.961 | 0.444 | 0.989 | 1.250 | 0.969 | 0.665 |
| **Mean** | **1.923** | **1.921** | **1.553** | **1.307** | **0.909** | **0.984** |
| **SD** | **1.344** | **0.792** | **0.397** | **0.362** | **0.160** | **0.252** |

The unit of lactate exchange ability is /min. vV̇O_2peak_ running speed at peak oxygen consumption

**Supplemental Table 2** The individual changes in velocity constant representing lactate removal ability (γ_2_, /min) for each running speed completed relative to peak oxygen consumption (vV̇O_2peak_)

| **Participant** | **vV̇O_2peak_** | **vV̇O_2peak_+2** | **vV̇O_2peak_+4** | **vV̇O_2peak_+6** | **vV̇O_2peak_+8** | **vV̇O_2peak_+10** |
| --- | --- | --- | --- | --- | --- | --- |
| 01 | 0.582 | 0.134 | 0.251 | 0.101 | 0.094 | N/A |
| 02 | 0.107 | 0.236 | 0.071 | 0.076 | 0.059 | N/A |
| 03 | 0.225 | 0.086 | 0.102 | 0.054 | N/A | N/A |
| 04 | 0.126 | 0.068 | 0.086 | 0.059 | N/A | N/A |
| 05 | 0.679 | 0.091 | 0.196 | 0.040 | N/A | N/A |
| 06 | 0.058 | 0.068 | 0.114 | 0.053 | N/A | N/A |
| 07 | 0.026 | 0.077 | 0.063 | 0.074 | N/A | N/A |
| 08 | 0.239 | 0.322 | 0.072 | 0.105 | N/A | N/A |
| 09 | 0.377 | 0.099 | 0.093 | 0.077 | N/A | N/A |
| 10 | 0.294 | 0.418 | 0.080 | 0.069 | 0.048 | 0.040 |
| 11 | 0.082 | 0.235 | 0.087 | 0.062 | 0.048 | N/A |
| 12 | 0.912 | 0.105 | 0.102 | 0.101 | 0.037 | 0.034 |
| 13 | 0.171 | 0.156 | 0.095 | 0.074 | 0.049 | N/A |
| 14 | 0.112 | 0.439 | 0.294 | 0.173 | 0.069 | 0.083 |
| **Mean** | **0.285** | **0.181** | **0.122** | **0.080** | **0.058** | **0.053** |
| **SD** | **0.255** | **0.125** | **0.069** | **0.032** | **0.018** | **0.022** |

The unit of lactate removal ability is /min. vV̇O_2peak_ running speed at peak oxygen consumption

**Supplemental Table 3** The individual changes in quantity of lactate accumulated (QLaA, mmol) for each running speed completed relative to peak oxygen consumption (vV̇O_2peak_)

| **Participant** | **vV̇O_2peak_** | **vV̇O_2peak_+2** | **vV̇O_2peak_+4** | **vV̇O_2peak_+6** | **vV̇O_2peak_+8** | **vV̇O_2peak_+10** |
| --- | --- | --- | --- | --- | --- | --- |
| 01 | 119.17 | 118.90 | 141.66 | 218.20 | 283.16 | - |
| 02 | 92.16 | 176.91 | 159.27 | 261.52 | 396.00 | - |
| 03 | 100.18 | 105.21 | 193.19 | 287.17 | - | - |
| 04 | 113.69 | 113.69 | 228.71 | 312.28 | - | - |
| 05 | 143.05 | 148.80 | 234.24 | 251.94 | - | - |
| 06 | 62.35 | 97.93 | 115.36 | 193.70 | - | - |
| 07 | 91.31 | 143.33 | 151.34 | 217.31 | - | - |
| 08 | 85.57 | 135.77 | 126.51 | 178.00 | - | - |
| 09 | 101.65 | 124.79 | 148.67 | 206.81 | - | - |
| 10 | 133.73 | 131.58 | 197.41 | 229.17 | 352.31 | 512.50 |
| 11 | 162.18 | 122.20 | 144.68 | 292.81 | 456.18 | - |
| 12 | 163.29 | 140.41 | 159.33 | 275.53 | 294.71 | 461.91 |
| 13 | 119.53 | 126.84 | 208.34 | 266.40 | 461.43 | - |
| 14 | 74.91 | 139.10 | 149.89 | 187.60 | 222.44 | 323.54 |
| **Mean** | **111.63** | **130.39** | **168.47** | **241.32** | **352.32** | **432.65** |
| **SD** | **29.64** | **19.06** | **35.91** | **41.16** | **84.15** | **79.87** |

The unit of quantity of lactate accumulated is mmol. vV̇O_2peak_ running speed at peak oxygen consumption

**Supplemental Table 4** The individual changes in blood lactate concentrations at the onset of recovery (La(0), mmol/L) for each running speed completed relative to peak oxygen consumption (vV̇O_2peak_)

| **Participant** | **vV̇O_2peak_** | **vV̇O_2peak_+2** | **vV̇O_2peak_+4** | **vV̇O_2peak_+6** | **vV̇O_2peak_+8** | **vV̇O_2peak_+10** |
| --- | --- | --- | --- | --- | --- | --- |
| 01 | 1.53 | 1.25 | 1.11 | 0.28 | 0.73 | - |
| 02 | 1.54 | 0.83 | 1.18 | 0.95 | 0.80 | - |
| 03 | 1.38 | 1.27 | 0.78 | 1.27 | - | - |
| 04 | 1.36 | 1.12 | 1.60 | 1.51 | - | - |
| 05 | 1.01 | 1.21 | 1.40 | 1.18 | - | - |
| 06 | 1.09 | 0.89 | 0.83 | 1.36 | - | - |
| 07 | 1.60 | 1.58 | 1.49 | 1.86 | - | - |
| 08 | 1.16 | 1.26 | 0.79 | 1.16 | - | - |
| 09 | 1.12 | 1.34 | 1.35 | 0.86 | - | - |
| 10 | 1.59 | 1.65 | 0.83 | 1.55 | 1.39 | 0.65 |
| 11 | 1.97 | 1.26 | 0.75 | 0.72 | 0.55 | - |
| 12 | 1.67 | 1.29 | 1.57 | 1.05 | 1.15 | 2.48 |
| 13 | 1.32 | 1.51 | 1.12 | 1.30 | 1.30 | - |
| 14 | 0.87 | 1.62 | 1.37 | 0.82 | 0.72 | 0.40 |
| **Mean** | **1.37** | **1.29** | **1.16** | **1.13** | **0.95** | **1.18** |
| **SD** | **0.29** | **0.24** | **0.30** | **0.38** | **0.30** | **0.93** |

The unit of blood lactate concentration at the onset of recovery is mmol. vV̇O_2peak_ running speed at peak oxygen consumption
